# Machine-learning classifiers based on non-enhanced computed tomography radiomics to differentiate anterior mediastinal cysts from thymomas and low-risk from high-risk thymomas: A multi-center study

**DOI:** 10.3389/fonc.2022.1043163

**Published:** 2022-11-24

**Authors:** Lan Shang, Fang Wang, Yan Gao, Chaoxin Zhou, Jian Wang, Xinyue Chen, Aamer Rasheed Chughtai, Hong Pu, Guojin Zhang, Weifang Kong

**Affiliations:** ^1^ Department of Radiology, Sichuan Academy of Medical Sciences and Sichuan Provincial People’s Hospital, Affiliated Hospital of University of Electronic Science and Technology of China, Chengdu, China; ^2^ Department of Radiology, Chinese Academy of Sciences Sichuan Translational Medicine Research Hospital, Chengdu, China; ^3^ Department of Radiology, Ningxia Hui Autonomous Region People’s Hospital, Yinchuan, China; ^4^ Department of Radiology, The First People’s Hospital of Liangshan Yi Autonomous Prefecture, Xichang, China; ^5^ Department of diagnostic imaging School of Computer Science, Nanjing University of Science and Technology, Nanjing, China; ^6^ Department of Diagnostic Imaging, Computed Tomography (CT) Collaboration, Siemens Healthineers, Chengdu, China; ^7^ Section of Thoracic Imaging, Cleveland Clinic Health System, Cleveland, OH, United States

**Keywords:** computed tomography, radiomics, thymoma, mediastinal cyst, machine learning (ML), classifier

## Abstract

**Background:**

This study aimed to investigate the diagnostic value of machine-learning (ML) models with multiple classifiers based on non-enhanced CT Radiomics features for differentiating anterior mediastinal cysts (AMCs) from thymomas, and high-risk from low risk thymomas.

**Methods:**

In total, 201 patients with AMCs and thymomas from three centers were included and divided into two groups: AMCs *vs.* thymomas, and high-risk vs low-risk thymomas. A radiomics model (RM) was built with 73 radiomics features that were extracted from the three-dimensional images of each patient. A combined model (CM) was built with clinical features and subjective CT finding features combined with radiomics features. For the RM and CM in each group, five selection methods were adopted to select suitable features for the classifier, and seven ML classifiers were employed to build discriminative models. Receiver operating characteristic (ROC) curves were used to evaluate the diagnostic performance of each combination.

**Results:**

Several classifiers combined with suitable selection methods demonstrated good diagnostic performance with areas under the curves (AUCs) of 0.876 and 0.922 for the RM and CM in group 1 and 0.747 and 0.783 for the RM and CM in group 2, respectively. The combination of support vector machine (SVM) as the feature-selection method and Gradient Boosting Decision Tree (GBDT) as the classification algorithm represented the best comprehensive discriminative ability in both group. Comparatively, assessments by radiologists achieved a middle AUCs of 0.656 and 0.626 in the two groups, which were lower than the AUCs of the RM and CM. Most CMs exhibited higher AUC value compared to RMs in both groups, among them only a few CMs demonstrated better performance with significant difference in group 1.

**Conclusion:**

Our ML models demonstrated good performance for differentiation of AMCs from thymomas and low-risk from high-risk thymomas. ML based on non-enhanced CT radiomics may serve as a novel preoperative tool.

## 1 Introduction

With the increasing application of chest CT imaging in clinical practice and lung cancer screening, the incidental detection of anterior mediastinal lesions among asymptomatic patients has become more frequent, with a reported prevalence of 0.73 – 0.9% (1, 2). The most common anterior mediastinal lesions are thymomas and anterior mediastinal cysts (AMCs), including thymic, bronchogenic, and pericardial cysts. Surgery or tissue acquisition is required for thymomas, while AMCs usually do not require treatment unless complications occur (3). However, AMCs are often misdiagnosed as thymomas, and the rate of non-therapeutic thymectomy is 22 – 68% (3–5). This results in a waste of medical resources and may expose patients with AMCs to surgical risks that could be avoided. Therefore, accurately distinguishing AMCs from thymomas before treatment is critical.

According to the World Health Organization (WHO) 2015 classification, thymomas can be subdivided into low-risk (types A, AB, and B1) and high-risk (types B2 and B3) groups depending on prognosis (6). Compared to the low-risk group, the high-risk group of thymomas is more likely to invade locally, has a smaller opportunity for complete surgical resection, may require multimodal therapy, and has higher tumor recurrence rates and mortality rates (7). Hence, it is crucial to accurately distinguish thymomas subtypes before treatment.

Contrast-enhanced CT (CECT) is the standard modality for diagnosis of anterior mediastinal lesions (8). The diagnostic accuracy of CECT for AMCs is 46% (9) because AMCs may also exhibit high attenuation (9) and pseudo-enhancement (10), especially in small lesions. The value of CT features for predicting the histologic subtype of thymomas remains unclear (9, 11, 12), which could be due to different enhancement time, with reported scan times of 30 – 90 seconds. Further, artifacts of contrast media may cause unstable and different image quality. CECT may also result in additional radiation dose and risk of contrast agent allergy. MRI is another option for AMCs and thymomas (13) but is expensive, requires long scanning times, and is not readily available, precluding its widespread use. In addition, CECT and MRI rely on the experience of the radiologist. In contrast, non-enhanced CT (NECT) image quality is stable and lacks artifacts and risk of contrast media. NECT is easily and widely available in clinical and screening at most hospitals. Therefore, there is a need to build radiomics-based ML models based on NECT images to identify AMCs and thymomas and to distinguish between subtypes of thymomas.

Radiomics has the potential to detect specific disease characteristics that cannot be visualized with current medical imaging modalities by quantitatively analyzing digital images. Qualitative CT radiomics analysis and radiomics-based ML have been applied for differential diagnosis of AMCs and thymomas in several studies (14, 15) and in risk prediction for thymomas (16–21), highlighting their potential for use in clinical practice to facilitate diagnosis and guide decision-making. However, previous studies have several limitations. First, no study to date has analyzed AMCs and thymomas, and the subtypes of thymomas were analyzed using the same ML model concurrently, with a focus on distinguishing the two types of lesions. This may lack applicability due to the complex and elusive conditions in clinical practice. Further, these studies were conducted based on images from one center, the sample sizes in several studies were small (14, 16), and the results lacked generalizability. Of note, radiomics studies generally extract a large number of features. Redundant features are more easily prone to over-fitting, and the choice of model classifiers should be a trade-off between computational burden and model efficacy (22–24) such that the appropriate feature section algorithm and model classifier are critical for ML model performance. Multiple image-based ML models have been used for diagnostic prediction of lesions located in the anterior skull (25). However, no study to date has used multiple ML models for distinguishing AMCs and thymomas, which may underestimate ML performance.

Therefore, this study aimed to investigate the value of multiple ML models (combinations of five feature selection algorithms and seven model classifiers) in preoperative differentiation of thymomas between AMCs and subtypes of thymomas based on multi-center NECT images.

## 2 Methods

### 2.1 Patient allocation

**Figure 1:**
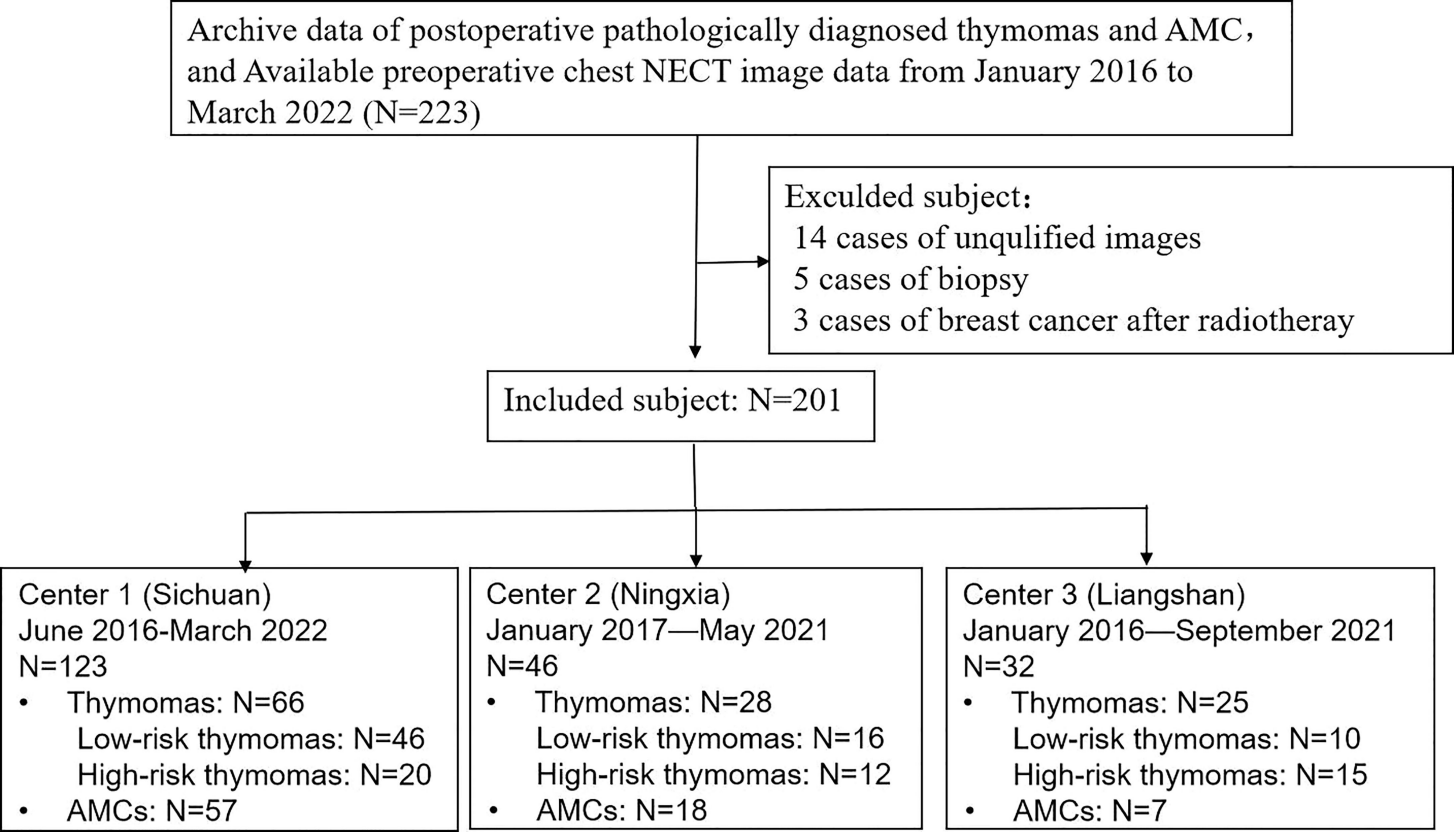
Flow diagram of subject inclusion.

This study was approved by the local ethics committee of the three participating hospitals, and the requirement for informed consent was waived. We included patients who underwent surgical resection and were pathologically confirmed with AMCs and thymomas between January 2016 and March 2022. Three different institutions (Center 1: Sichuan Provincial People’s Hospital, Center 2: Ningxia Hui Autonomous Prefecture People’s Hospital, and Center 3: The First People’s Hospital of Liangshan Yi Autonomous Prefecture) provided the cases, in consideration of better validation of the built model. Inclusion criteria were as follows (1): pathologically diagnosed with AMCs and thymomas; and (2) underwent NECT imaging within 4 weeks before surgery; and (3) available clinical data and surgical records. Exclusion criteria were: (1) poor image quality due to artifacts or other reasons; (2) previous biopsy or treatment before CT scans because the biopsies had the potential to cause incorrect typing due to the specificity of thymomas (19). A final total of 201 patients were enrolled in our study, and 22 patients were excluded. A flow diagram of the patient selection process is shown in **Figure 1**.

Clinical information assessment included the following: age; sex; symptoms including myasthenia gravis (MG), chest pain, respiratory symptoms (cough and dyspnea); and other symptoms. This information was collected by a junior radiologist based on clinical records.

### 2.2 Image acquisition and interpretation

CT was performed with three dual-source CT scanners (Somatom Definition Flash, Drive, and Force; Siemens Medical Solutions, Germany). All three institutions used the same scanning protocols. The acquisition parameters were 0.5 mm, 0.5 mm, and 0.625 mm detector collimation; 120 kVp tube voltage; 0.5 s gantry rotation time; 1 mm, 1 mm, and 1.5 mm reconstructed section thickness; and 0.8 mm, 0.8 mm, and 1 mm reconstruction intervals.

The area from the thoracic inlet caudally, including the adrenal glands, was scanned. Chest NECT images were obtained from the Picture Archiving and Communication Systems (PACS) database of the three centers. All NECT images were reviewed by two thoracic radiologists, who had more than 10 years of experience in chest CT study interpretation and were blinded to the histopathological results and clinical information. If there was variation in the results, the two radiologists reviewed the CT images together. Any discrepancies were resolved by discussion until consensus was reached. Subjective image findings included average CT value, lesion size (maximum, minimum, and average diameter on axial images, maximum diameter on sagittal or coronal images, and ratio of maximum to minimum diameter), density uniformity, calcification, shape (round, oval, and irregular), and margin (circumscribed, lobulated, and spiculated), vascular invasion, pericardial effusion, plural effusion, lymph node enlargement (short diameter >1 cm).

Given the difference in recommended treatments for the cases, we divided the lesions into two groups: group 1 for discrimination of AMCs from thymomas, and group 2 for discrimination of low-risk from high-risk thymomas. Two thoracic radiologists recorded their first diagnosis for each patient in two groups, that is, AMCs or thymomas in group 1 and low-risk or high-risk thymomas in group 2.

### 2.3 Segmentation and feature extraction

Segmentation of the entire lesion was performed using LIFEx software (version 6.3, http://www.lifexsoft.org) by two experienced thoracic radiologists (readers 1 and 2, with 15 and 13 years of experience in chest CT study interpretation, respectively) who were blinded to pathology results. The 3D volume-of-interest (VOI) manual segmentation was performed separately for 40 randomly chosen images by both readers.

**Figure 2:**
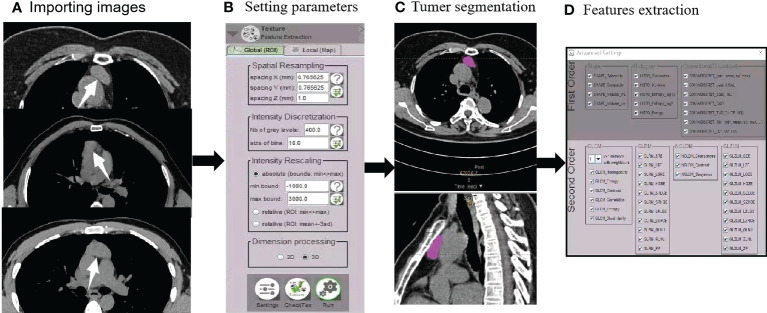
Radiomics flowchart. **(A)** After importing images (AMCs, low-risk thymomas, and high-risk thymomas), **(B)** automated settings of radiomics feature parameters were performed. **(C)** Tumor segmentation contours were manually drawn in the 3D VOI. **(D)** First- and second-order features were extracted.

A total of 73 features were obtained from two orders, including first-order features from shape-based matrix and histogram-based matrix, and second-order/higher-order features from gray-level co-occurrence matrix (GLCM), gray-level zone length matrix (GLZLM), neighborhood gray-level dependence matrix (NGLDM), and gray-level run length matrix (GLRLM). A flowchart of radiomics feature extraction is shown in **Figure 2**.

The consistency of features from different machines and inter-observer reproducibility of texture features were assessed using intra- and inter-class correlation coefficients (ICCs). An ICCs > 0.75 is considered good agreement.

### 2.4 Feature selection and model classifiers

In consideration of the high-dimensional nature of the features that may contain non-informative or redundant predictors, optimal features were first selected for the predictive mode. Different feature selection methods, including Least Absolute Shrinkage and Selection Operator (LASSO) regression (17, 19, 21) and Gradient Boosting Decision Tree (GBDT) have been reported to be appropriate for high-dimensional data analysis (18). To address this issue, five supervised feature selection methods were separately employed, including GBDT, Extreme Gradient Boosting (Xgboost), Random Forest (RF), Distance Correlation, and LASSO. The methods were performed separately with the same aim of identifying a subset of predictors to increase accuracy and simultaneously reduce model complexity. A similar problem also required resolution with regard to the selection of model classifiers. After selecting the five subsets of predictors, we separately used seven model classifiers with each of the subsets as inputs based on our data. These seven classifiers included Support Vector Machine (SVM), K-Nearest Neighbor (KNN), Linear Discriminant Analysis (LDA), GaussianNB, Adaboost, Logistic Regression (LR), and Decision Tree (DT).

### 2.5 Model construction and validation

We contrasted two models based on the enrolled factors: a radiomics model (RM) based on NECT image radiomics features alone, and a combined model (CM) based on the combination of clinical information, NECT subjective findings, and image radiomics features together for the classification tasks. Models of both types, comprising 35 discriminative ML models with RM and CM, were used for group 1 and group 2 discrimination tasks.

Patients were randomly divided into training and testing groups at a ratio of 9:1. We used resampling methods and 10-fold cross-validation to estimate the performance and generalization ability of the models using our training cohorts. The discriminative performance of different models was quantified as area under the receiver operating characteristic (ROC) curve (AUC), accuracy, sensitivity, and specificity.

### 2.6 Statistical analysis

Statistical analyses of clinical characteristics and subjective CT findings were performed using SPSS (Version 22.0, IBM Corp. Armonk, NY, USA). Continuous variables were summarized using the mean ± standard deviation, and categorical variables were presented as counts or percentages. Comparisons between groups was used a student’s *t*-test, Mann-Whitney *U* test and Chi-square test as required. ML algorithms were programmed with Python Programming Language and scikit-learn packages. Receiver operating characteristic (ROC) curve was used to evaluate the performance of the ML model, and the area under the curve (AUC), accuracy, sensitivity and specificity were calculated. The DeLong test was used to compare the AUCs of the RMs and CMs with 35 ML models in both groups. All statistical tests were two-sided. *P*-values < 0.05 were considered statistically significant.

## 3 Results

### 3.1 General patient characteristics

In total, 82 patients with AMCs and 119 patients with thymomas were included in the study. The 82 cases of AMCs comprised 20 thymic, 32 bronchogenic, and 30 pericardial cysts. The 119 patients with thymomas comprised 72 patients with low-risk thymomas (13 type A, 35 type AB, and 24 type B1) and 47 patients with high-risk thymomas (26 type B2 and 21 type B3).

Between the AMCs and thymomas group, we observed significant differences in symptoms (more cases of MG in patients with thymomas, *P*=0.001), average CT value (higher in patients with thymomas, *P*<0.001), maximum and minimum diameter on axial images (longer in patients with thymomas, *P*<0.001 and *P*<0.001, respectively), and ratio of max to min diameter (longer in patients with AMCs, *P*=0.007). Significantly more patients with density uniformity, calcification, round shape, spiculated margin, pleural effusion, and lymph node enlargement was observed in patients with thymomas than in patients with AMCs (*P*<0.001, *P*=0.023, *P*=0.014, *P<*0.001, *P*=0.017, and *P*=0.039, respectively). No significant differences were observed in sex, age, long size on sagittal or coronal images, pericardial effusion, and vascular invasion (*P*>0.05). Between low-risk and high-risk thymomas, we observed significant differences in maximum and minimum diameter on axial images (longer for low-risk thymomas, *P*=0.01, *P*<0.001, respectively); Significantly more patients with spiculated margin and lymph node enlargement were observed for high-risk than for low-risk thymomas (*P*<0.001, *P*=0.024, respectively). No significant differences were noted in sex, age, symptoms, average CT value, long size on sagittal or coronal images, ratio of long diameter to short diameter, density uniformity, calcification, and shape, vascular invasion, pericardial effusion, pleural effusion (*P*>0.05). The characteristics of patients and lesions of two groups are compared in **Table 1**.

**Table 1:** Clinical characteristics of the patients and features of the lesions.

Patients	AMC group, N=82	Thymomas, N=119	*P*-value	Low risk thymomas, N=72	High risk thymomas, N=47	*P*-value
**Sex, (％)**			0.935			0.247
Male	37 (45.1%)	54.02 (44.5%)		29 (40.3%)	24 (51.1%)	
Female	45 (54.9%)	66 (55.5%)		43 (59.7%)	23 (48.9%)	
**Age,** years			0.831			0.839
Mean ± SD	53.66 ± 10.57	54.02 ± 12.43		55.14 ± 12.38	52.30 ± 12.43	
Range	31-77	21-73		19-78	22-71	
**Symptoms, (％)**			0.001			0.398
Myasthenia gravis	2 (2.4%)	24 (20.2%)		13 (18.1%)	11 (23.4%)	
Chest pain	4 (4.9%)	13 (10.9%)		10 (13.9%)	3 (6.4%)	
Respiratory symptoms (cough, dyspnea)	11 (13.4%)	12 (10.1%)		9 (12.5%)	3 (6.4%)	
Other symptom	9 (11%)	13 (10.9%)		6 (8.3%)	7 (14.9%)	
NO symptom	56 (68.3%)	57 (47.9%)		34 (47.2%)	23 (48.9%)	
**Average CT value, HU,** mean ± SD,	26.83 ± 20.94	41.33 ± 11.15	< 0.001	40.38 ± 12.57	42.79 ± 8.46	0.130
**Size,** mean ± SD,						
maximum diameter on axial image (cm)	3.44 ± 2.23	4.83 ± 2.32	< 0.001	4.99 ± 2.61	4.60 ± 1.80	0.010
minimum diameter on axial image (cm)	2.35 ± 1.46	3.25 ± 1.54	< 0.001	3.44 ± 1.749	2.96 ± 1.09	< 0.001
Long size on sagittal or coronal image (cm)	4.62 ± 2.66	5.43 ± 2.38	0.943	5.71 ± 2.57	5.00 ± 2.01	0.051
Ratio of max to min diameter *	2.07 ± 0.58	1.84 ± 0.61	0.007	1.82 ± 0.63	1.87 ± 0.58	0.624
**Density uniformity, (％)**			< 0.001			0.098
yes	66 (80.5%)	41 (34.5%)		29 (40.3%)	12 (25.5%)	
no	16 (19.5%)	78 (65.5%)		43 (59.7%)	35 (74.5%)	
**Calcification, (％)**			0.023			0.174
yes	10 (12.2%)	30 (25.2%)		15 (20.8%)	15 (31.9%)	
no	72 (87.8%)	89 (74.8%)		57 (79.2%)	32 (68.1%)	
**Shape, (％)**			0.014			0.619
round	13 (15.9%)	40 (33.6%)		26 (36.1%)	14 (29.8%)	
oval	28 (34.1%)	37 (31.1%)		23 (31.9%)	14 (29.8%)	
irregular	41 (50%)	42 (35.3%)		23 (31.9%)	19 (40.4%)	
**Margin, (％)**			< 0.001			< 0.001
Circumscribed	43 (52.4%)	25 (21%)		22 (30.6%)	3 (6.4%)	
Lobulated	28 (34.1%)	35 (29.4%)		24 (33.3%)	11 (23.4%)	
Spiculated	11 (13.4%)	59 (49.6%)		26 (36.1%)	33 (70.2%)	
**Vascular invasion, (％)**			0.238			0.078
yes	0 (0%)	2 (1.7%)		0 (0%)	2 (4.3%)	
no	82 (100%)	117 (98.3%)		72 (100%)	45 (95.7%)	
**Pericardial effusion, (％)**			0.217			0.702
yes	7 (8.5%)	17 (14.3%)		11 (15.3%)	6 (12.8%)	
no	75 (91.5%)	102 (85.7%)		61 (84.7%)	41 (87.2%)	
**Pleural effusion, (％) **			0.017			0.905
yes	0 (0%)	8 (6.7%)		5 (6.9%)	3 (6.4%)	
no	82 (100%)	111 (93.3%)		67 (93.1%)	44 (93.6%)	
**Lymph node, (％) enlargement**			0.039			0.024
yes	0 (0%)	6 (5%)		1 (1.4%)	5 (10.6%)	
no	82 (100%)	113 (95%)		71 (98.6%)	42 (89.4%)	

SD, standard deviation.

Of the 82 patients with AMCs, radiologists made a correct first-choice diagnosis in 29 (35.4%) cases based on NECT images, however, 53 (64.6%) cases of AMCs were misdiagnosis as thymomas. Of the 119 patients with thymomas, 5 (4.2%) patients, including 3 patients with type B1 and 2 patients with type B2 thymomas, were misdiagnosed as having AMCs by radiologists, and underwent surgical resection as these patients presented with clinical symptoms. The radiologists correctly diagnosed 42 (58.3%) of the 72 low-risk thymoma cases, and 31 (66%) of the 47 high-risk thymomas. The AUC, accuracy, sensitivity, and specificity of radiologist were 0.656, 0.716, 0.966, and 0.354 for thymomas in group 1, and 0.626, 0.613, 0.660, and 0.583 for high-risk thymomas in group 2.

### 3.2 Inter-operator and inter-machine consistency

Two chest radiologists defined the lesions by VOIs. Each feature was calculated twice for each lesion. The ICCs ranged from 0.814 to 0.923, indicating high inter-operator consistency in obtaining the features. Finally, we randomly selected one of the results for ML analysis. The reproducibility of the radiomics features by the different machines was satisfactory (ICCs ranged from 0.786 to 0.912).

### 3.3 ML model performance

In total, 35 discriminative ML models through the combination of five selection methods and seven classifiers for two types of RMs and CMs were used for two group discrimination tasks. Among two group

#### 3.3.1 Group 1: ML model performance for discriminating AMCs from thymomas

Among 35 RMs, 32 RMs had AUCs > 0.7, and 16 RMs had AUCs > 0.8. In particular, QBDT, Xgboost, and RF as the feature selection method with SVM, LDA, KNN, and GausiannNB as classifiers exhibited higher value of ROCs. Among models, RF+SVM had the highest AUC of 0.878, but the sensitivity of this model was only 0.586. GBDT+SVM had a high AUC and good balance between sensitivity and specificity; thus, this model was considered the best discriminate model. The AUC, accuracy, sensitivity, and specificity of GBDT+SVM were 0.876, 0.806, 0.670, and 0.90 in the testing group, and 0.909, 0.818, 0.70, and 0.90 in the training group, respectively. RF+KNN, Xgboost + LDA, and GBDT+GaussianNB had AUCs ≥ 0.847 in the testing group, indicating reliable diagnostic performance. Details of model performance are presented in **Table 2**.

**Table 2:** Results of discriminative RMs in group 1 in the testing group.

	QBDT		Xgboost		RF		Distance correlation		LASSO	
	Accuracy	AUC	Accuracy	AUC	Accuracy	AUC	Accuracy	AUC	Accuracy	AUC
SVM	0.806	0.876	0.761	0.841	0.761	0.878	0.761	0.779	0.722	0.787
KNN	0.776	0.823	0.766	0.832	0.771	0.853	0.761	0.804	0.731	0.751
LDA	0.771	0.814	0.772	0.849	0.781	0.829	0.767	0.730	0.752	0.819
GausiannNB	0.761	0.847	0.746	0.824	0.721	0.831	0.756	0.799	0.601	0.740
Adaboost	0.731	0.828	0.761	0.819	0.716	0.769	0.701	0.740	0.711	0.786
LR	0.771	0.829	0.757	0.791	0.751	0.796	0.757	0.715	0.697	0.775
DT	0.711	0.706	0.756	0.746	0.721	0.714	0.672	0.658	0.682	0.668

AUC, Area under curve; Decision tree, DT; GBDT, Gradient boosting decision tree; KNN, K-nearest neighbor; LASSO, least absolute shrinkage and selection operator; LDA, Linear Discriminant analysis; LR, Logistic regression; RF, Random forest; SVM, Support vector machine; Xgboost, Extreme gradient boosting.

**Figure 3:**
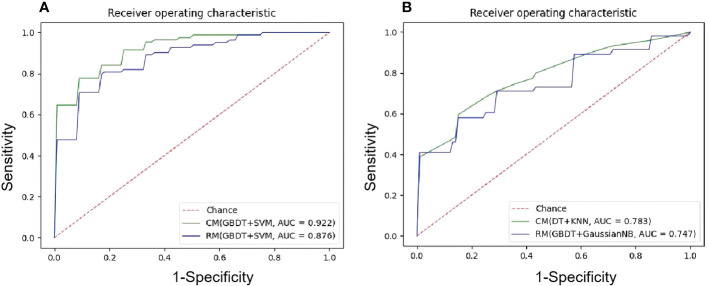
The ROC curves of the best ML models. **(A)** NECT-based RM and CM with(GBDT+SVM) in the testing sets in group1, mean AUC of 0.876 and 0.922 were presented; **(B)** NECT-based RM with (GBDT+GaussianNB) and CM with (DT+KNN) in the testing tests in group 2, mean AUC of 0.747 and 0.783 were presented.

Among 35 CMs, 34 CMs had AUCs > 0.7, and 19 CMs had AUCs > 0.8. The combination of QBDT and Xgboost as the feature selection method with SVM and GausiannNB as classifiers exhibited better diagnostic performance, similar to RMs. Among them, GBDT+SVM demonstrated the best diagnostic performance, with the highest AUC and a good balance between sensitivity and specificity. The AUC, accuracy, sensitivity, and specificity of this model were 0.922, 0.850, 0.81, and 0.882 in the testing group and 0.963, 0.903, 0.87, and 0.92 in the training group, respectively. Other models including GBDT+GausiannNB, GBDT+KNN, GBDT+LR, and Xgboost+SVM also demonstrated reliable diagnostic performance in CMs in group 1, with AUCs ≥ 0.894 in the testing group. Moreover, of 35 CMs, 28 had higher value of AUCs than those for RMs in the testing group, with statistically significant differences for the combination of GBDT+KNN (*P*=0.015), GBDT+GausiannNB (*P*=0.0328), and Xgboost+LR (*P*=0.0389). Details of model performance are presented in **Table 3**. The ROC curve of the best RM and CM models in group 1are presented in **Figure 3**.

**Table 3:** Results of discriminative CMs in group 1 in the testing group.

	QBDT		Xgboost		RF		Distance Correlation		LASSO	
	Accuracy	AUC	Accuracy	AUC	Accuracy	AUC	Accuracy	AUC	Accuracy	AUC
SVM	0.850	0.922	0.821	0.894	0.815	0.882	0.751	0.777	0.722	0.787
KNN	0.835	0.908	0.810	0.882	0.840	0.882	0.746	0.778	0.731	0.751
LDA	0.884	0.825	0.869	0.806	0.867	0.791	0.732	0.737	0.752	0.819
GausiannNB	0.830	0.913	0.815	0.872	0.736	0.848	0.752	0.785	0.601	0.740
Adaboost	0.811	0.882	0.721	0.807	0.776	0.822	0.721	0.753	0.711	0.786
LR	0.845	0.903	0.811	0.886	0.791	0.860	0.757	0.715	0.697	0.775
DT	0.801	0.798	0.826	0.818	0.761	0.759	0.722	0.708	0.682	0.668

AUC, Area under curve; Decision tree, DT; GBDT, Gradient boosting decision tree; KNN, K-nearest neighbor; LASSO, least absolute shrinkage and selection operator; LDA, Linear Discriminant analysis; LR, Logistic regression; RF, Random forest; SVM, Support vector machine; Xgboost, Extreme gradient boosting.

#### 3.3.2 Group 2: Model performance for discriminating low-risk from high-risk thymomas

Among 35 RMs, 14 models had AUCs > 0.7. Among them, GBDT+LR and GBDT+SVM had the highest AUCs of 0.763 and 0.760, respectively, but with sensitivities of 0.41 and 0.46, respectively. Hence, these models were unable to meet the thresholds for clinical application. The GBDT+GaussianNB model exhibited a good balance between sensitivity and specificity and was considered the best discriminate model. The AUC, accuracy, sensitivity, and specificity for this model were 0.747, 0.638, 0.580, and 0.679 in the testing group and 0.818, 0.724, 0.688, and 0.747 in the training group, respectively. Details of model performance are presented in **Table 4**.

**Table 4:** Results of discriminative RMs in group 2 in the testing group.

	QBDT		Xgboost		RF		Distance correlation		LASSO	
	Accuracy	AUC	Accuracy	AUC	Accuracy	AUC	Accuracy	AUC	Accuracy	AUC
SVM	0.695	0.760	0.645	0.702	0.636	0.684	0.655	0.699	0.605	0.570
KNN	0.628	0.658	0.639	0.634	0.611	0.669	0.654	0.655	0.545	0.502
LDA	0.705	0.730	0.679	0.721	0.611	0.702	0.696	0.740	0.620	0.663
GausiannNB	0.638	0.747	0.689	0.717	0.628	0.733	0.604	0.721	0.546	0.576
Adaboost	0.687	0.666	0.630	0.638	0.595	0.598	0.637	0.599	0.605	0.582
LR	0.696	0.763	0.680	0.732	0.670	0.722	0.688	0.720	0.664	0.587
DT	0.613	0.602	0.637	0.630	0.608	0.570	0.561	0.532	0.546	0.524

AUC, Area under curve; Decision tree, DT; GBDT, Gradient boosting decision tree; KNN, K-nearest neighbor; LASSO, Least absolute shrinkage and selection operator; LDA, Linear Discriminant analysis; LR, Logistic regression; RF, Random forest; SVM, Support vector machine; Xgboost, Extreme gradient boosting.

Among 35 CMs, 19 of all 35 models had AUCs > 0.7, and 2 models had AUCs > 0.8. Among models, the combination of GBDT+GaussianNB, GBDT+SVM, and RF+SVM had the highest AUCs of 0.808, 0.802, and 0.797, respectively, but the sensitivities were only 0.520, 0.58, and 0.55, respectively, in the testing group. The Distance correlation+KNN model had a good balance between sensitivity and specificity; thus, this model was considered the best discriminate model. The AUC, accuracy, sensitivity, and specificity of this model were 0.783, 0.774, 0.665, and 0.848 in the testing group and 0.891, 0.815, 0.70, and 0.89 in the training group, respectively. Distance Correlation+LDA with an AUC of 0.783 also exhibited reliable diagnostic performance in group 2. Moreover, Of all 35 CMs, 28 had higher value of AUCs than those of RMs in the testing group, but the difference was not statistically significant (*P*=0.09-1.0, *P*>0.05). Details of model performance are presented in **Table 5**. The ROC curve of the best RM and CM models in group 1are presented in **Figure 3**.

**Table 5:** Results of discriminative CMs in group 2 in the testing group.

	QBDT		Xgboost		RF		Distance correlation		LASSO	
	Accuracy	AUC	Accuracy	AUC	Accuracy	AUC	Accuracy	AUC	Accuracy	AUC
**SVM**	0.721	0.802	0.723	0.799	0.721	0.797	0.731	0.761	0.605	0.570
**KNN**	0.730	0.756	0.656	0.726	0.730	0.777	0.774	0.783	0.545	0.502
**LDA**	0.687	0.785	0.637	0.686	0.687	0.778	0.755	0.793	0.620	0.663
**GausiannNB**	0.705	0.808	0.723	0.774	0.688	0.772	0.680	0.752	0.546	0.576
**Adaboost**	0.637	0.676	0.688	0.697	0.596	0.610	0.645	0.612	0.605	0.582
**LR**	0.705	0.774	0.714	0.783	0.730	0.780	0.705	0.787	0.664	0.587
**DT**	0.640	0.629	0.631	0.616	0.630	0.621	0.630	0.597	0.546	0.524

AUC, Area under curve; Decision tree, DT; GBDT, Gradient boosting decision tree; KNN, K-nearest neighbor; LASSO, least absolute shrinkage and selection operator; LDA, Linear Discriminant analysis; LR, Logistic regression; RF, Random forest; SVM, Support vector machine; Xgboost, Extreme gradient boosting.

### 3.4 Feature selection

The selection method of integrated GBDT exhibited higher value of AUCs compared to the others for the same model algorithms in both groups. The features selected by the best discrimination models in the two groups are presented in **Table 6**. LASSO as a feature selection method did not select any clinical information and subjective CT findings for analysis in CMs in both groups. As a result, the diagnostic ability of RMs and CMs did not change.

**Table 6:** Features selected by the GBDT in the two groups.

Group 1: AMCs to thymomas		Group 2: Low-risk to high-risk thymomas	
NECT-based RM;	NECT-based CM;	NECT-based RM;	NECT-based CM;
N=8	N=11	N=15	N=12
CONVENTIONAL_HU min	CONVENTIONAL_HUQ2	CONVENTIONAL_HU min	CONVENTIONAL_HUQ2
CONVENTIONAL_HU max	SHAPE_Sphericity onlyFor3DROI)	CONVENTIONAL_HUQ2	SHAPE_Sphericity only For 3D ROI)
CONVENTIONAL_HUQ2	GLCM_Correlation	DISCRETIZED_HUQ2	SHAPE_Surface (mm2) only For 3D ROI
CONVENTIONAL_HUQ3	GLRLM_LGRE	SHAPE_Sphericity only For 3D ROI)	SHAPE_Compacity onlyFor3DROI
SHAPE_Sphericity only For 3D ROI)	CONVENTIONAL_HUmin	SHAPE_Surface(mm2) only For 3D ROI	GLCM_Correlation
SHAPE_Surface (mm2) only For 3D ROI	Symptoms	SHAPE_Compacity only For 3D ROI	GLRLM_SRLGE
GLCM_Correlation	Average CT value	GLCM_Energy =Angular Second Moment	GLZLM_HGZE
GLZLM_LZLGE	Maximum diameter on axial image(cm)	GLCM_Correlation	GLZLM_GLNU
	Ratio of max diameter to min diameter	GLRLM_SRLGE	Average CT value
	Density uniformity	GLRLM_SRHGE	Minimum diameter on axial image(cm)
	Margin	NGLDM_Contrast	Lymph node enlargement
		GLZLM_HGZE	Margin
		GLZLM_SZHGE	
		GLZLM_GLNU	
		GLZLM_ZLNU	
		DISCRETIZED_HUQ2	

CM, combined model; GLCM, gray-level co-occurrence matrix; GLZLM, gray-level zone length matrix; GLRLM, gray-level run length matrix; NGLDM, neighborhood gray-level dependence matrix; RM, radiomics model.

## 4 Discussion

In this study, we proposed ML discriminative models based on a multi-center dataset of NECT images to differentiate AMCs from thymomas and low-risk from high-risk thymomas. Further, we aimed to identify the optimal discriminative models from the combination of five feature selection methods and seven classifiers. Since the features we selected were based on multi-center NECT images and clinical findings, these ML models have the potential to be used as a convenient and non-invasive discrimination tool to predict the risk of AMCs and thymomas, which has implications for treatment decisions.

### 4.1 Discrimination of AMCs and thymomas

In our study, the diagnostic performance of radiologists was unsatisfactory towards distinguishing AMCs from thymomas, with an AUC of 0.656 and low specificity. Almost 2/3 patients with AMCs were misdiagnosed as thymomas, and the possibility of thymomas was not ruled out for the remaining 1/3 AMCs. Hence, the surgeons adopted more aggressive surgical resection for all AMCs patients, who should avoid unnecessary surgeries. This highlights the need to identify radiomics tools to improve the diagnostic accuracy of AMCs, despite the benign nature and low incidence of these lesions.

Few studies have aimed to distinguish AMCs and thymomas using CT imaging radiomics features (14, 15). Yasaka et al. (14) reported that solid anterior mediastinal masses could be differentiated from cysts using quantitative CT texture analyses with AUC of 0.869 in 76 patients with NECT. However, the sample size of the study was small. Liu et al. (15) reported that the combined models, including radscore and CT value, exhibited better diagnostic performance compared to radscore models for distinguishing AMCs and type B1 and B2 thymomas, with AUCs of 0.928 and 0.856 based on NECT, and 0.938 and 0.899 based on CECT in the testing group, respectively. However, in clinical practice, the subtypes of thymomas cannot be easily distinguished from each other, and this study used only one ML model.

Our study demonstrated that both RMs and CMs based on NECT had outstanding diagnostic performance for distinguishing AMCs and thymomas. 80% CMs had higher value of AUC than RMs, however only several CMs exhibited significant differences. Then using the combination of GDBT+SVM, which was the optimal comprehensive performance ML model, the AUCs for RM and CM were 0.876 and 0.922, respectively, but the difference between RM and CM was not statistically significant. These results were similar to the findings of Liu et al. (15).

The RMs based on NECT could improve diagnostic accuracy of AMCs from thymomas compared to the radiologist’s diagnosis based on CT images. This suggests that radiomics has the potential to detect specific characteristics of AMCs and thymomas that cannot be visualized by radiologists. Moreover, CMs based on NECT slightly improved the diagnostic performance using few combinations. The results indicate that clinical and CT subjective features are important for the differential diagnosis. However, CT findings are determined by subjective judgment and may be influenced by the radiologist’s diagnostic experience, and clinical and CT subjective features played a limited role in the differential diagnosis of AMCs and thymomas. In contrast, Radiomics features objectively reflect the internal structural heterogeneity of lesions, so that RMs based on NECT showed the potential to be a relatively simple, objective, and efficient diagnostic tool.

### 4.2 Discrimination of low-risk and high-risk thymomas

The diagnostic performance of radiologists to distinguish high-risk from low-risk thymomas had an AUC of 0.626. GBDT+GaussianNB in RM and Distance correlation+KNN in CM exhibited good diagnostic performance for discriminating low-risk and high-risk thymomas, with AUCs of 0.747 and 0.783, respectively. GBDT+SVM also had good AUCs of 0.760 and 0.802 in RM and CM, respectively. But no significant differences were found between RMs and CMs.

Several studies (16–19) have attempted to identify subtypes of thymomas using imaging radiomics and ML models. Liu et al. (18) established triple-classification RMs, corresponding clinical, and clinical-semantic RMs, based on NECT images with TETs. The AUCs of the RM, clinical model, and clinical-sematic RM were 0.686, 0.787, and 0.770 for low-risk thymomas; 0.601, 0.699, and 0.689 for high-risk thymomas; and 0.632, 0.689, and 0.783 for thymic carcinoma, respectively, in the validation set, using Sperman + GBDT as feature section method and LR as ML classifier. The AUCs for low-risk thymomas and high-risk thymomas in our study were higher than those reported by Liu et al. (18). We speculated that these differences may be mainly due to the different classifications. In this regard, the results of three classifications RM are lower than those of two classifications RM, which is shown in Yin et al’s study (26). Another reason may be that we used multiple ML models and identified the optimal ML models for our data, while Liu’s study used only one ML classifier, which may have underestimated the efficiency.

Chen et al. (19) built three RMs and CMs based on venous-phase CECT images and reported high diagnostic performance for predicting the invasiveness of thymic epithelial tumors, with AUCs of 0.944 and 0.953, respectively. However, no significant difference was observed between RM and CM. The AUCs in our study were lower than those in Chen et al.’s study. Several reasons may underpin these differences. One reason is differences in study subjects, whereby thymic carcinoma was included in the high-risk group in Chen et al.’s study. It has been reported that thymic carcinoma has obvious infiltration characteristics on CT and is easy to identify (27). The ML models in Chen et al.’s study was based on CECT venous-phase images, which has the potential to provide more feature information.

All three studies demonstrated that CMs have higher AUC values than those of RMs, based on NECT or CECT images. However, the differences were not statistically significant in our study or the study done by Chen et al. We could not make comparisons to Liu’s study as the statistical analyses were unavailable. We speculated that texture features are reflective of the heterogeneity of lesions and play a dominant role in the differentiation of subtypes of thymomas. Additionally, clinical information and subjective CT findings may be helpful for differential diagnosis of the different subtype of thymomas, which should be verified with more studies.

### 4.3 Feature selection algorithms

Among five feature selection algorithms, GBDT exhibited the best performance in both groups. GBDT is an iterative decision tree algorithm consisting of multiple decision trees. The conclusions of all trees cumulatively arrived at a final answer. It was first proposed as an algorithm with greater generalization (28). Liu et al. (18) demonstrated that the GBDT algorithm performed stably in thymomas radiomics feature extraction, although their study did not have the same number of radiomics features as that in our study.

LASSO as a feature selection algorithm was most widely used alone or combined with variance threshold, Select K Best in imaging radiomics modeling (17, 19, 21). In our study, LASSO performed well with moderate discrimination in both groups. The same ROCs were observed between RMs and CMs because no clinical information or subjective CT finding features were selected by LASSO in CMs in both groups. LASSO is based on the penalty method for variable selection of sample data. Through compression of the original coefficients, the originally small coefficients are directly compressed to 0, such that the corresponding variables of these coefficients are regarded as non-significant variables, and the insignificant variables are directly discarded. In addition, among 34 CMs, only several CMs performed significantly better than RMs in groups 1, no CMs performed better than RMs in group 2. These results are consistent with each other, and demonstrated that clinical information and subjective CT finding features did not play a significant role in differentiating AMCs and thymomas and subtypes of thymomas.

The three other feature selection algorithms were first used in diagnostic models of AMCs and thymomas and also exhibited reliable performance, which can be applied and verified in bigger sample data sets.

### 4.4 ML model classifiers

Among seven model classifiers (LDA, SVM, Adaboost, KNN, Gaussian NB, LR, and DT), SVM was the top-performing classifier in both groups. Gaussian NB, KNN, and LR exhibited reliable discrimination efficiency. SVM is an optimal boundary classification method based on VC dimension theory and structural risk minimization criterion with maximum interval classifier in feature space. The purpose of SVM is to find a compromise value that minimizes the empirical risk and confidence interval. While concurrently considering training error and model complexity, it can obtain better classification effects when the number of samples is small (29).

Another study (21) used six model classifiers to differentiate high-risk from low-risk thymomas based on CECT images and reported that the top three classifiers were KNN, LR and SVM, with AUCs of 0.943, 0.943, and 0.857, respectively. These three classifiers also performed reliably in our study. In previous studies on ML for thymomas, LR as a model classifier was most widely used in imaging radiomics modeling (18–20), indicating stable diagnostic performance. Gausiann NB was first used in diagnostic models of thymomas and also exhibited reliable performance.

We can see from these studies. Because each classifier has its own characteristics and application scope, it is necessary to select the most appropriate classifier for different data sets. In order to maximize the performance of ML, it is necessary to try multiple classifiers for one data set.

### 4.5 Study limitations

Similar to other retrospective studies, there may have been selection bias in this study. This study did not perform external validation due to the low incidence of thymoma and small sample size. In the future, we intend to collect data from more centers for analysis. Further, this study did not build the ML models with CECT images because of the different contrast time (25-90 s) in the three centers. In this regard, CECT images may provide more feature information. Future studies should compare the NECT and CECT ML models with same scanning parameters. Finally, due to the complex and elusive conditions in clinical application, it is necessary to make three classifications under the same subgroup to distinguish AMCs, low-risk, and high-risk thymomas, which is one of our future research intentions.

## 5 Conclusion

This study proposed ML predictive models based on NECT radiomics and clinical parameters *via* multiple combinations of selection algorithms and ML classifiers. Our study demonstrated that ML models could distinguish thymomas from AMCs with a high diagnostic performance and distinguish subtypes of thymomas with a moderate performance. The combined models of clinical information, subjective CT findings, and radiomics features slightly improved performance compared to RMs alone. Our prediction model has the potential for application in clinical practice as a new and convenient tool.

## Data availability statement

The raw data supporting the conclusions of this article will be made available by the authors, without undue reservation.

## Ethics statement

The studies involving human participants were reviewed and approved by ethics committee of Sichuan provincial people’s hospital. Written informed consent for participation was not required for this study in accordance with the national legislation and the institutional requirements.

## Author contributions

LS, FW, YG, GZ, WK: Conceptualization and design. LS, FW, YG, WK: Methodology, investigation, formal analysis, validation and writing - original draft. CZ: Investigation and formal analysis. JW, XC: Methodology and statistical analysis. WK, AC: Writing - review and editing. GZ, HP: Supervision and project administration. All authors contributed to the article and approved the submitted version.

## Funding

This work was supported by the Science and Technology Department of Sichuan Province Research Project (No. 2022YFS0075), the Sichuan Provincial Cadre Health Research Project (No. 2020-225, 2022-208), and the Sichuan Academy of Medical Sciences & Sichuan Provincial People’s Hospital Research Fund (No. 2022QN25).

## Conflict of interest

The authors declare that the research was conducted in the absence of any commercial or financial relationships that could be construed as a potential conflict of interest.

## Publisher’s note

All claims expressed in this article are solely those of the authors and do not necessarily represent those of their affiliated organizations, or those of the publisher, the editors and the reviewers. Any product that may be evaluated in this article, or claim that may be made by its manufacturer, is not guaranteed or endorsed by the publisher.

## Glossary

**Table:**

AMC	anterior mediastinal cyst
AUC	area under the curve
CECT	contrast-enhanced computed tomography
CM	combined model
CT	computed tomography
DT	Decision Tree
GBDT	Gradient Boosting Decision Tree
GLCM	gray-level co-occurrence matrix
GLRLM	gray-level run length matrix
GLZLM	gray-level zone length matrix
KNN	k-Nearest Neighbor
LASSO	least absolute shrinkage and selection operator
LDA	Linear Discriminant Analysis
LR	logistic regression
NECT	non-enhanced computed tomography
NGLDM	neighborhood gray-level dependence matrix
PACS	Picture Archiving and Communication Systems
RF	random forest
RM	radiomics model
ROC	receiver operating characteristic
SVM	Support Vector Machine
SD	Standard Deviation
VOI	volume-of-interest
WHO	World Health Organization
Xgboost	Extreme Gradient Boosting
